# Aphasia-specific or generic outcomes? a comparison of two health-related quality of life instruments for economic evaluations of aphasia treatments

**DOI:** 10.1007/s11136-025-04040-8

**Published:** 2025-07-26

**Authors:** Sally Zingelman, Sarah J. Wallace, Joosup Kim, Sam Harvey, Miranda L. Rose, John E. Pierce, Kathleen L. Bagot, Dominique A. Cadilhac

**Affiliations:** 1 Queensland Aphasia Research Centre, School of Health and Rehabilitation Sciences, The University of Queensland, Herston, Australia; 2 Surgical Treatment and Rehabilitation Service (STARS), Education and Research Alliance, The University of Queensland and Metro North Health, Herston, Australia; 3 Centre of Research Excellence in Aphasia Recovery and Rehabilitation, La Trobe University, Melbourne, Australia; 4 Department of Medicine, Stroke and Ageing Research, School of Clinical Sciences at Monash Health, Monash University, Clayton, Australia; 5 Stroke Division, The Florey Institute of Neuroscience and Mental Health, Heidelberg, Australia; 6 School of Allied Health, Human Services and Sport, La Trobe University, Melbourne, Australia

**Keywords:** Economic evaluation, Patient reported outcome measures, Health-related quality of life, Aphasia, EQ-5D, SAQOL-39g

## Abstract

**Purpose:**

Economic evaluations based on health-related quality of life (HRQOL) inform healthcare decisions. The generic EuroQol 5-Dimensions Health Questionnaire, 3-Level (EQ-5D-3L) permits conversion to utility values required for economic evaluations but is not validated for people with aphasia. The aphasia-specific Stroke and Aphasia Quality of Life Scale-39 g (SAQOL-39g) measures HRQOL, however, cannot be used to generate utility values. This study aimed to compare the performance of these two instruments.

**Methods:**

HRQOL was rated at baseline and 12 weeks in participants of the Constraint Induced or Multi-Modal Personalised Aphasia Rehabilitation (COMPARE) randomised controlled trial. We assessed: (1) distribution of self-rated HRQOL scores, (2) convergent validity between EQ-5D-3L (domains; utility values; visual analogue scale) and SAQOL-39g (domain scores; total mean scores) using Spearman’s correlations, (3) Construct validity through exploratory factor analysis, and (4) discriminative ability of converted EQ-5D-3L utilities in measuring compromised HRQOL (SAQOL-39g scores ≤ 4).

**Results:**

Participants (*n* = 201 baseline, *n* = 190 12 weeks) completed both instruments (69% male, median age 63.6 years, median time since stroke 2.5 years). Ceiling effects were high for the EQ-5D-3L at baseline (45–79%) versus the SAQOL-39g (0–6%). Convergent validity between the SAQOL-39g communication domain and the EQ-5D-3L (*r* = 0.04–0.28) was weak at both time points. Factor analysis revealed distinct underlying constructs between instruments. EQ-5D-3L utility scores demonstrated reasonable performance (0.80 baseline; 0.78 12-weeks) in measuring poor HRQOL.

**Conclusion:**

Our findings suggest that EQ-5D-3L use in economic evaluations including people with aphasia requires caution. Alternative HRQOL instruments require evaluation to ensure fair prioritisation of aphasia treatments.

**Supplementary Information:**

The online version contains supplementary material available at 10.1007/s11136-025-04040-8.

## Abstract (plain language summary)

Up to 40% of patients with stroke experience aphasia, a communication disorder significantly impacting health-related quality of life. Economic evaluations are critical to guide resource allocation to treatments that show the greatest health benefits to patients and value to society. We compared two questionnaires measuring health-related quality of life. One questionnaire, the EQ-5D-3L has a scoring system adapted for use in economic evaluations. The second questionnaire, the SAQOL-39g, does not have an adapted scoring system, but was designed to measure elements of quality of life relevant to people with aphasia.

Results were from a randomised control trial of 201 people with long-term aphasia. Participants were mostly men with a median age of 63.9 years. Many participants reported no problems with their health on the EQ-5D-3L before treatments, but few did so on the SAQOL-39g. We propose that the EQ-5D-3L may lack sensitivity to measure improvements following treatments in people with mild to moderate aphasia. Our analysis showed the EQ-5D-3L and SAQOL-39g measure different parts of health-related quality of life: the SAQOL-39g alone measures communication, the EQ-5D alone measures pain/discomfort, and both measure physical functioning and psychosocial well-being. These results are important for economic evaluations using the EQ-5D-3L in populations with aphasia. To ensure economic evaluations are meaningful and aphasia treatments can be reliably evaluated, there is a need evaluate alternative questionnaires that 1-have scoring systems adapted for use in economic evaluations and 2- measure communication-related elements of quality of life that are relevant to people with aphasia.

**Supplementary Information:**

The online version contains supplementary material available at 10.1007/s11136-025-04040-8.

## Introduction

Aphasia is a common acquired language disorder that affects a person’s ability to communicate effectively and is experienced by approximately 40% of patients with stroke [1]. Aphasia can have a profound impact on a person’s Health-Related Quality of Life (HRQOL), a multidimensional construct that quantifies the impact of health conditions and treatments on an individual’s functioning and participation [2]. Aphasia can make it difficult for people to express themselves, participate in social activities, maintain relationships, and remain independent [3]. Subsequently, people with aphasia may experience frustration, social isolation, and diminished psychosocial well-being [4]. Significant advancements are being made in developing aphasia treatment approaches which aim to improve HRQOL. However, to promote the implementation of effective interventions, there is a need to establish reliable methods to evaluate the cost-effectiveness of aphasia treatments [2, 5].

Economic evaluations assess the value of health care treatments to inform resource allocation decisions in healthcare [6]. The most cost-effective options can be identified by comparing the costs and outcomes of alternative treatments. Utility values are numerical measures that represent how society values living in each particular health state and are used to quantify these health outcomes. Utility values range between zero and one, with one signifying full health and zero indicating death. Scores may also fall below zero, indicating a patient reported a health condition that is perceived worse than death [2, 7]. Generic patient-reported instruments which can be converted into utility values as part of their scoring system are widely used in economic evaluations [7]. When the utility scores are combined with the quantity of life gained from treatments, a Quality Adjusted Life Year (QALY) is produced [8]. A QALY represents a treatment’s impact on a patient’s overall well-being and is the measure of ‘benefit’ relative to costs, commonly used as a health outcome in economic evaluations. QALYs are commonly used as a health outcome as they are designed to enable comparisons across different health conditions and treatments through the use of standardised measurement instruments [2, 8].

Internationally, the EuroQol-5 Dimensions Health Questionnaire 3 Level (EQ-5D-3L) is one of the most widely used generic instruments [9, 10]. Generic instruments are those which include domains of HRQOL relevant to most health conditions. While generic instruments facilitate comparisons across health conditions, the EQ-5D-3L does not include a communication domain, which is a highly relevant aspect of HRQOL for people with aphasia [5]. Instead, patients are asked to rate problems performing usual activities such as ‘work, study, housework, family, or leisure activities’, that involve communication skills [9, p. 9]]. Whether these ratings correlate with communication-related items on other instruments is unknown. Evidence for the psychometric properties of the EQ-5D-3L in stroke populations is limited. In a systematic review analysing 19 EQ-5D-3L studies, Cameron et al. (2021) reported test-retest validity was limited (one study of fair quality; intraclass coefficient or weighted Kappa ≥ 0.70) while moderate support was evaluated for construct validity and responsiveness (consistent findings across multiple studies of fair methodological quality) [11]. To our knowledge, no authors have evaluated the measurement properties of the EQ-5D-3L against a validated HRQOL instrument for patients with post-stroke aphasia.

Investigators evaluating aphasia treatments customarily use validated condition-specific instruments to measure HRQOL as a treatment outcome [12, 13]. The Stroke and Aphasia Quality of Life Scale-39 g (SAQOL-39g) was developed as a patient-reported outcome measure that captures the domains of HRQOL relevant to people with aphasia, including communication ability [14, 15]. The SAQOL-39g scoring does not produce utility values and, therefore, cannot be used to calculate QALYs [5, 15]. The SAQOL-39g has robust psychometric properties, with strong internal consistency (Cronbach’s alpha 0.74–0.94), test-retest reliability (intraclass correlation coefficient 0.89–0.98) and good construct validity (correlations between sub-domains 0.50–0.80) [15].

The aim of this study was to compare the performance of the generic EQ-5D-3L to the aphasia- specific SAQOL-39g to provide guidance for the EQ-5D-3L’s use, or not, in future economic evaluations of aphasia treatments. Specifically, the aims were to understand how well the EQ-5D-3L and the SAQOL-39g are correlated, and the sensitivity and specificity of the EQ-5D-3L to classify HRQOL as measured by the SAQOL-39g. If scores between the EQ-5D-3L and the SAQOL-39g are highly correlated, this means that the EQ-5D-3L may be an appropriate HRQOL instrument for use in economic evaluations which include people with aphasia. However, if scores are not correlated, further research may be required to explore alternatives that can produce valid utility values for economic evaluation.

## Materials and methods

### Study design and data source

This secondary analysis used deidentified, repeated measures data from the Constraint Induced or Multi-Modal Personalised Aphasia Rehabilitation (COMPARE) randomised controlled trial. The COMPARE trial was approved by institutional (La Trobe University) and hospital (Gold Coast University Hospital and participating hospital sites) ethics committees [16]. Participants were eligible if they had aphasia resulting from a stroke occurring at least six months prior to consent (Western Aphasia Battery-Revised Aphasia Quotient (WAB-R AQ) scores of < 93.8), medical stability, pre-stroke English fluency, independent toileting and a significant other available for assessments [16]. Exclusion criteria included previous non-stroke neurological events, severe apraxia of speech or dysarthria, major clinical depression or other mental health condition, and uncorrected sensory or other serious medical condition potentially impacting or influencing study participation [17]. Analysis for the current study included 201 COMPARE participants who completed the EQ-5D-3L and SAQOL-39g instruments at baseline [16]. Participants with incomplete outcome data or lost to follow-up were excluded from the 12-week analysis as utility values and total mean scores required complete the EQ-5D-3L or SAQOL-39g questionnaire responses.

COMPARE participants were recruited through direct community advertising and 22 hospitals in Australia and New Zealand (July 2016 to March 2021). From 342 people screened, 249 met the eligibility criteria and 216 were randomised to one of three treatment groups based on aphasia severity (WAB-R AQ scores) and location: constraint induced aphasia therapy- plus (n*n* = 71, 33%), multi-modality aphasia therapy (*n* = 75, 35%) and usual care (*n* = 71, 32%). Post-randomisation, 15 participants withdrew (three dropouts, nine due to treatment dissatisfaction, three due to COVID-19 restrictions), resulting in 201 participants completing baseline data collection [16]. Detailed recruitment, and withdrawal information is published elsewhere [16–18]. Reporting of this study followed the Strengthening the Reporting of Observational Studies in Epidemiology (STROBE) statement [19].

### Quality of life instruments

The EQ-5D-3L measures HRQOL across five dimensions (mobility, self-care, usual activities, pain/discomfort and anxiety/depression) with three response levels: 1 = no problems, 2 = some problems, or 3 = extreme problems [9]. Patient responses create a five-digit health state profile matched to established country-specific value sets, generating utility values from 0 (death) to 1 (perfect health) [7, 8]. Utility values are multiplied by the duration of time spent in that health state to estimate QALYs. The EQ-5D-3L includes a Visual Analogue Scale (VAS) where patients rate their overall health from 0 (worst imaginable) to 100 (best imaginable) [7].

The SAQOL-39g contains 39 items across physical (16 items), psychosocial (16 items) and communication (7 items) domains with five response options: 1 = couldn’t do it at all, 2 = a lot of trouble, 3 = some trouble, 4 = a little trouble and 5 = no trouble at all. Domain and overall mean scores (1–5) are calculated, with higher score indicating better HRQOL [15]. Scores of four or below may be indicative of compromised quality of life [3].

**Fig. 1:**
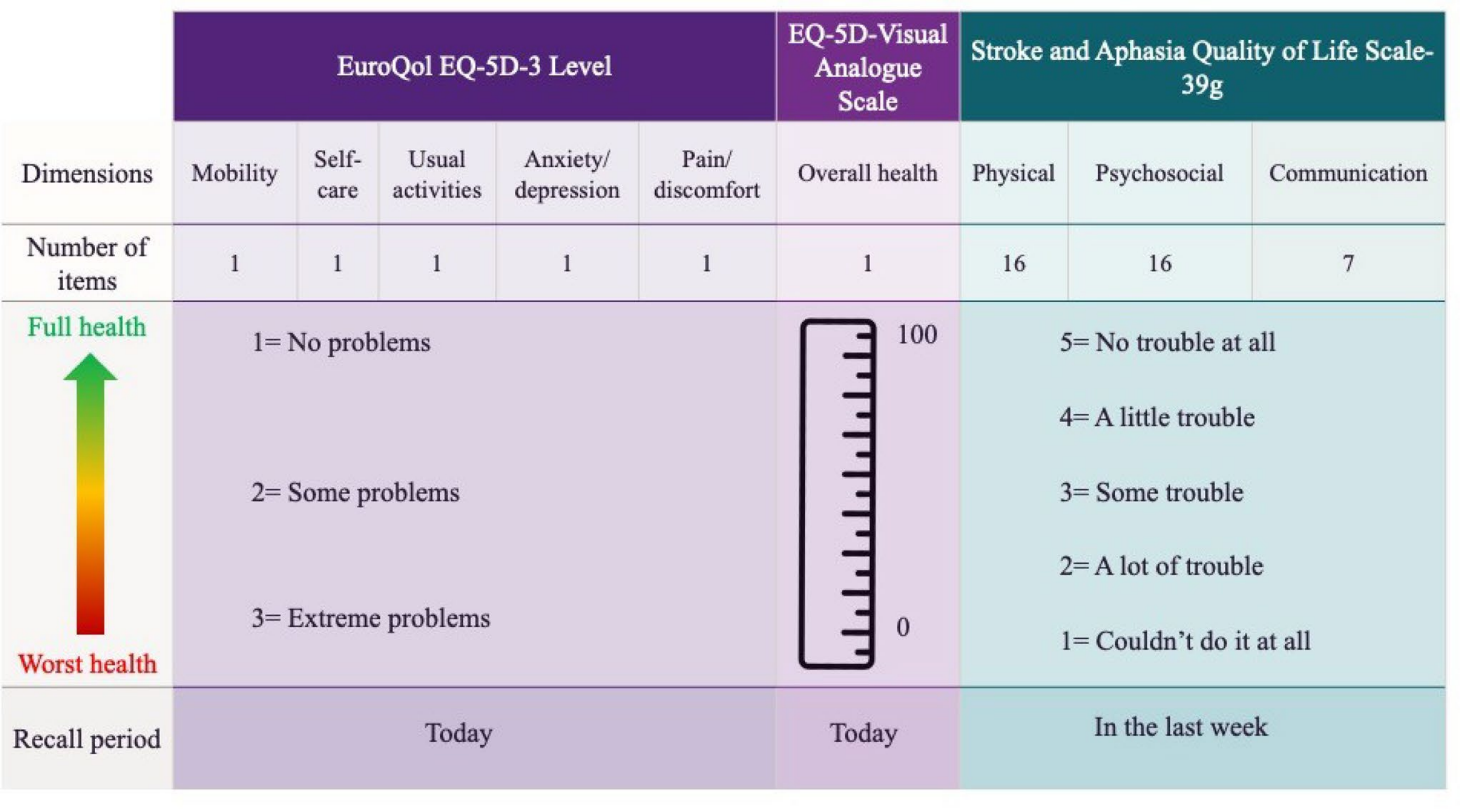
Comparison of features between the EQ-5D-3 Level and the Stroke and Aphasia Quality of Life Scale 39-g quality of life outcome measurement instruments

The two instruments differ substantially (Fig. 1). The EQ-5D-3L covers five dimensions aligning with physical, mental, and social health functioning [20], while the SAQOL-39g focuses on three domains which are affected by post-stroke aphasia, including communication. Recall periods for the two instruments differs (today or within the last week, respectively). Lower SAQOL-39g scores, EQ-5D-3L utility scores, and VAS scores indicate poorer health. However, for EQ-5D-3L item responses, the lowest score (1) indicates best perceived functioning, and the highest score (3) represents the worst perceived functioning for each domain [9, 15].

### Data availability

Data that support the findings of this study are held by third parties and are available upon reasonable request to the corresponding author of the COMPARE trial (author M.L.R).

### Ethics approval

Ethical approval for this project was obtained from the La Trobe Human Ethics Low Risk Committee (Approval number: HEC23119). Approval to use deidentified COMPARE trial data was obtained from the data custodian (author M.L.R, La Trobe University).

### Statistical analysis

Data were pooled for this analysis. Baseline participant characteristics were summarised using descriptive statistics. All other analyses were repeated in baseline and 12-week follow-up data. An algorithm validated in an Australian general population was used to convert baseline and follow-up EQ-5D-3L domain scores to utility values, as reported elsewhere [21]. Less than 10% of outcome data were missing, eliminating the need for data imputation [18]. Demographic characteristics of participants with missing versus complete baseline EQ-5D-3L and SAQOL-39g outcome data compared using chi-square tests for categorical variables and t-tests for continuous variables. Participant withdrawal is outlined alongside the COMPARE study results [18]. SAQOL-39g domain scores were reversed in the Spearman correlation and exploratory factor analyses for consistency of severity direction between the two measures. All analyses were conducted using STATA/SE 18.0 [22].

The distribution of scores at baseline and 12-week follow up were analysed with descriptive statistics and visualised with scatterplots for the EQ-5D-3L and SAQOL-39g. Convergent validity between the EQ-5D-3L and SAQOL-39g scores was explored by use of Spearman’s rank correlation coefficient. Correlation *r* values were interpreted as weak (0.1–0.39), moderate (0.4–0.69), high (0.7–0.89) or very high (0.9-1.0) per established benchmarks [23]. The discriminative ability of the EQ-5D-3L utility values and VAS was explored using a Receiver Operator Characteristic (ROC) curve analysis to examine how well they could classify participants according to their SAQOL-39g scores. We dichotomised SAQOL-39g scores at < 4 versus ≤ 4 for the ROC analysis, representing the lower half of the response scale (1–4 ‘couldn’t do it at all’ to ‘some trouble’ vs. the upper range (5: ‘no trouble at all’), based on the instrument developers’ description of the scores [3]. This cutoff point distinguishes between participants reporting some degree of difficulty (scores ≤ 4) versus those reporting no difficulties (score of 5) in HRQOL. Area Under the Curve (AUC) results range from 0.5 to 1. Given the exploratory nature of the analysis and the comparison between two subjective HRQOL measures, AUC values were described qualitatively rather than categorised using established diagnostic benchmarks. An exploratory factor analysis was conducted to understand the underlying structure of the EQ-5D-3L and explore construct validity in a population of people with aphasia. The number of factors retained was selected with reference to the Kaiser criterion, where factors with eigenvalues greater than one were retained. Uniqueness values ranged from 0 to 1, values of ≥ 0.6 were interpreted as high; with 40% or less of variance explained by common factors [24].

## Results

### Participant characteristics

Participants were predominately male (69%), aged between 55 and 70 years, with a median two and a half years since their stroke (Table 1). The majority of the cohort (94%) had mild or moderate aphasia (Table 1). At baseline, 201 participants completed both the EQ-5D-3L and SAQOL-39g (Table 2). Participants with missing baseline data were more likely to be aged over 70 years and have a self-reported nervous system disorder. No other participant characteristics differed significantly between groups.

**Table 1:** Characteristics of the study cohort who completed the EQ-5D-3L and SAQOL-39g at baseline and those who did not

Variable	Complete data (*n* = 201), n (%)	Missing data (*n* = 15), n (%)	*p* Value
Age			**0.030**
< 55 years	57 (28)	3 (20)	
55–70 years	88 (44)	3 (20)	
> 70 years	56 (28)	9 (60)	
Sex			0.461
Female	62(31)	6 (40)	
Male	139 (69)	9 (60)	
Years of full-time education			0.151
< 10 years	15 (8)	3 (20)	
10–15 years	94 (47)	8 (53)	
> 15 years	90 (45)	4 (27)	
Time post stroke			0.403
< 1 year	28 (14)	4(27)	
1–2 years	45 (22)	5(33)	
2–5 years	73 (36)	3(20)	
5–10 years	46(23)	2(13)	
> 10 years	9 (5)	1(7)	
Aphasia severity*			0.724
Severe	6 (3)	-	
Moderate	53 (26)	3(20)	
Mild	137 (68)	12(80)	
Above cut-off	5 (3)	-	
Language use			
Fluent English prior to stroke	201 (100)	15 (100)	
Self-reported Comorbidities			
Cardiovascular disease	200 (99)	15(100)	0.784
Nervous system disorder	78 (39)	1 (7)	**0.012**
Musculoskeletal disease	68 (34)	4 (27)	0.562
Psychiatric Disorder	64 (32)	6 (40)	0.515
Ophthalmological disease	34	1 (7)	0.299

*Western Aphasia Battery- Revised, Aphasia Quotient Scores. Severe (0–31.2); Moderate (31.3–62.5); Mild (62.6–93.6); Above cut-off (93.7–100)

**Table 2:** Distributions of scores for self-rated quality of life

EQ-5D-3L^*^	Mobility	Self-Care	Usual Activities	Anxiety/Depression		Pain/Discomfort	Utility Score	Visual Analogue Scale
Baseline (*n* = 201)								
No problems *n* (%)	117 (58)	156 (78)	91 (45)	151 (75)		118 (59)		
Some problems *n* (%)	84 (42)	43 (21)	98 (49)	49 (24)		80 (40)		
Extreme problems *n* (%)	0 (0)	2 (1)	12 (6)	3 (1)		3 (1)		
Mean	1.41	1.23	1.60	1.25		1.42	0.79	75.71
SD (95% CI)	0.49 (1.36, 1.50)	0.45 (1.16, 1.29)	0.60 (1.52, 1.69)	0.45 (1.19, 1.31)		0.52 (1.36, 1.51)	0.20 (0.76, 0.82)	17.31 (72.80, 77.82)
**12 weeks (** *n* ** = 190)**								
No problems *n* (%)	107 (56)	151 (80)	97 (51)	149 (78)		120 (63)		
Some problems *n* (%)	81 (43)	37 (19)	88 (46)	38 (20)		65 (34)		
Extreme problems *n* (%)	2 (1)	2 (1)	5 (3)	3 (2)		5 (3)		
Mean	1.44	1.21	1.51	1.23		1.30	0.79	82.55
SD (95% CI)	0.52 (1.37, 1.52)	0.44 (1.15, 1.27)	0.55 (1.44, 1.60)	0.46 (1.17, 1.30)		0.54 (1.32, 1.47)	0.21 (0.76, 0.83)	28.33 (73.71, 81.802)
**SAQOL-39g** ^+^	Physical		Psychosocial		Communication		Total	
**Baseline (** *n* ** = 201)**								
No trouble at all *n* (%)	12 (6)		2 (1)		0 (0)			
A little trouble *n* (%)	115 (57)		74 (37)		32 (16)			
Some trouble *n* (%)	53 (27)		79 (39)		79 (39)			
A lot of trouble *n* (%)	17 (8)		40 (20)		66 (33)			
Couldn’t do it at all *n* (%)	4 (2)		7 (3)		25 (12)			
Mean	4.09		2.00		3.59		3.69	
SD (95% CI)	0.80 (3.99, 4.21)		0.83 (3.46, 3.70)		0.83 (2.90, 3.14)		0.65 (3.60, 3.78)	
**12 weeks (** *n* ** = 190)**								
No trouble at all *n* (%)	15 (8)		3 (1)		2 (1)			
A little trouble *n* (%)	103 (54)		73 (39)		32 (17)			
Some trouble *n* (%)	52 (27)		68 (36)		87 (45)			
A lot of trouble *n* (%)	18 (10)		39 (20)		53 (28)			
Couldn’t do it at all *n* (%)	2 (1)		7 (4)		16 (9)			
Mean	4.08		3.59		3.14		3.71	
SD (95% CI)	0.81 (3.97, 4.20)		0.87 (3.47, 3.72)		0.81 (3.03, 3.27)		0.68 (3.62, 3.81)	

^*^EQ-5D-3L: Euroqol EQ-5D 3 Level. Score range 1 (no problems) to 3 (extreme problems). Utility score converted from domain scores using an algorithm validated in an Australian general population.

^#^VAS: Visual Analogue Scale. Score range 0 (worst health imaginable) to 100 (best health imaginable). ^+^SAQOL-39g: Stroke and Aphasia Quality of Life scale, 39 item. Score range 1 (Couldn’t do it at all) to 5 (No trouble at all)

### Differences in self-rated quality of life at baseline and 12-weeks

**Fig. 2:**
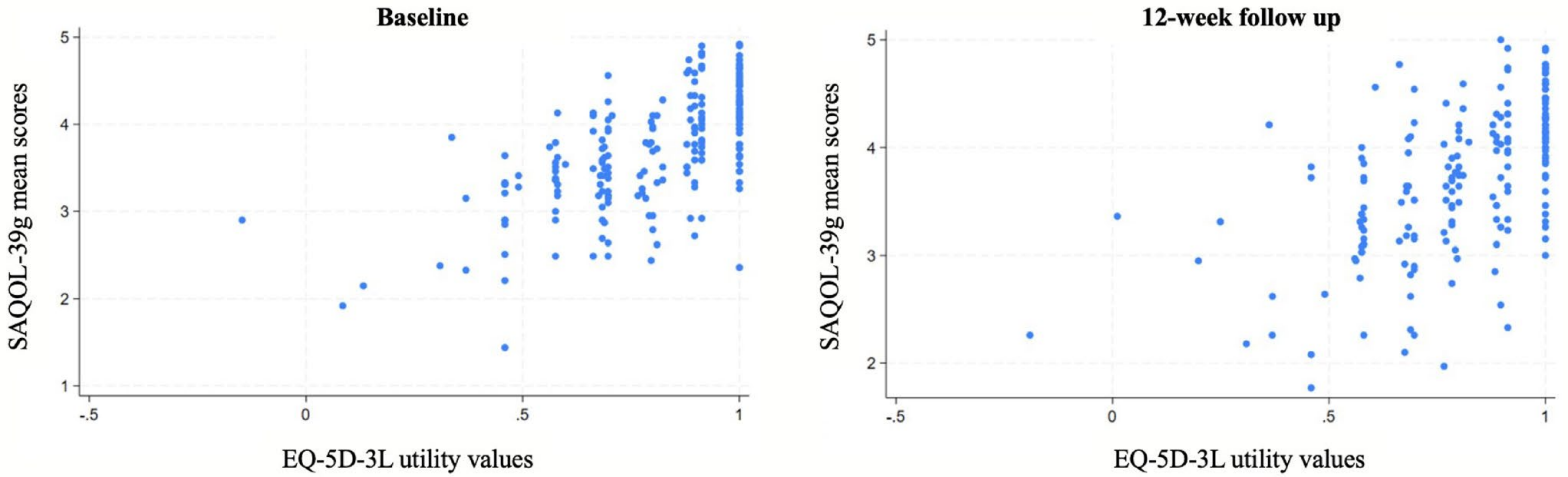
Scatterplots demonstrating the weak positive association between the EQ-5D-3L and SAQOL-39g at two time points

Participants reported minor problems with quality of life on the EQ-5D-3L utility scores (mean 0.79, SD 0.20), with just over a quarter reporting perfect health at baseline (utility value = 1, 26.4%, *n* = 53). SAQOL-39g scores (range: 3.0-4.09) indicated participants experienced minor problems across all domains of HRQOL, however, no participants reported ‘no trouble at all’ across all three domains at baseline (Table 2). Ceiling effects were observed on the EQ-5D-3L: At baseline, more than 50% of responders reported ‘no problems’ across all domains of HRQOL except the usual activities domain (45%; Table 2). This pattern was consistent at 12-week follow up, although the proportion of responders indicating ‘no problems’ on usual activities rose to 50%. On the SAQOL-39g, most responses were spread between the three middle categories (a little trouble, some trouble, a lot of trouble), with ‘no trouble at all’ being the least selected response category at baseline (7%) and 12 weeks (10%). Scatterplots demonstrated a weak positive association between the EQ-5D-3L and SAQOL-39g domain scores, with broadly dispersed data at both baseline and 12-week follow up. This pattern indicates a weak relationship between the two measures at both time points (Fig. 2).

### Convergent validity between EQ-5D-3L and SAQOL-39g scores

The communication domain of the SAQOL-39g demonstrated a consistently weak relationship with domain, index, and VAS scores on the EQ-5D-3L at both time points. The SAQOL-39g physical domain correlated moderately, except for the anxiety/depression domain which was weak. The SAQOL-39g psychosocial domain correlated weakly to the EQ-5D-3L domains, other than anxiety and depression and the utility values, which were moderate. The SAQOL-39g total mean score was moderately correlated to the EQ-5D-3L at baseline (*r *= 0.63) and 12-week follow up (*r* = 0.60). Overall, correlations were not found to be strong enough to enable the values on one measure to predict the values on the other (Table 3).

**Table 3:** Spearman’s correlations between baseline and 12-week follow up scores on the EQ-5D-3L and the SAQOL-39g

	Baseline EQ-5D-3L^*^						
**Baseline SAQOL-39g** ^**+**^	Mobility	Self-care	Usual activities	Anxiety/ depression	Pain/ Discomfort	Utility Value	VAS^#^
Physical	0.61	0.54	0.46	0.29	0.42	0.69	0.39
Psychosocial	0.30	0.11	0.31	0.40	0.36	0.44	0.34
Communication	0.15	0.15	0.20	0.23	0.14	0.26	0.21
Total Mean Score	0.47	0.37	0.43	0.41	0.31	0.63	0.44
	**12-week follow up EQ-5D-3L** ^*^						
**12-week follow up SAQOL-39g** ^**+**^	Mobility	Self-care	Usual activities	Anxiety/ depression	Pain/ Discomfort	Utility Value	VAS^#^
Physical	0.52	0.53	0.45	0.20	0.39	0.63	0.42
Psychosocial	0.27	0.17	0.20	0.43	0.32	0.45	0.42
Communication	0.12	0.12	0.15	0.25	0.04	0.23	0.28
Total Mean Score	0.42	0.39	0.34	0.38	0.37	0.60	0.46

*r*-value correlations: 0.1–0.39 weak, 0.4–0.69 moderate, 0.7–0.89 high, 0.9-1.0 very high

^*^ *EQ-5D-3L: Euroqol EQ-5D 3 Level.*

^*#*^ *VAS: Visual Analogue Scale.*

^*+*^*SAQOL-39g: Stroke and Aphasia Quality of Life scale*,* 39 item. SAQOL-39g domain scores were reversed for consistency of severity direction between the two measures*

### Discriminative ability of the EQ-5D-3L

**Fig. 3:**
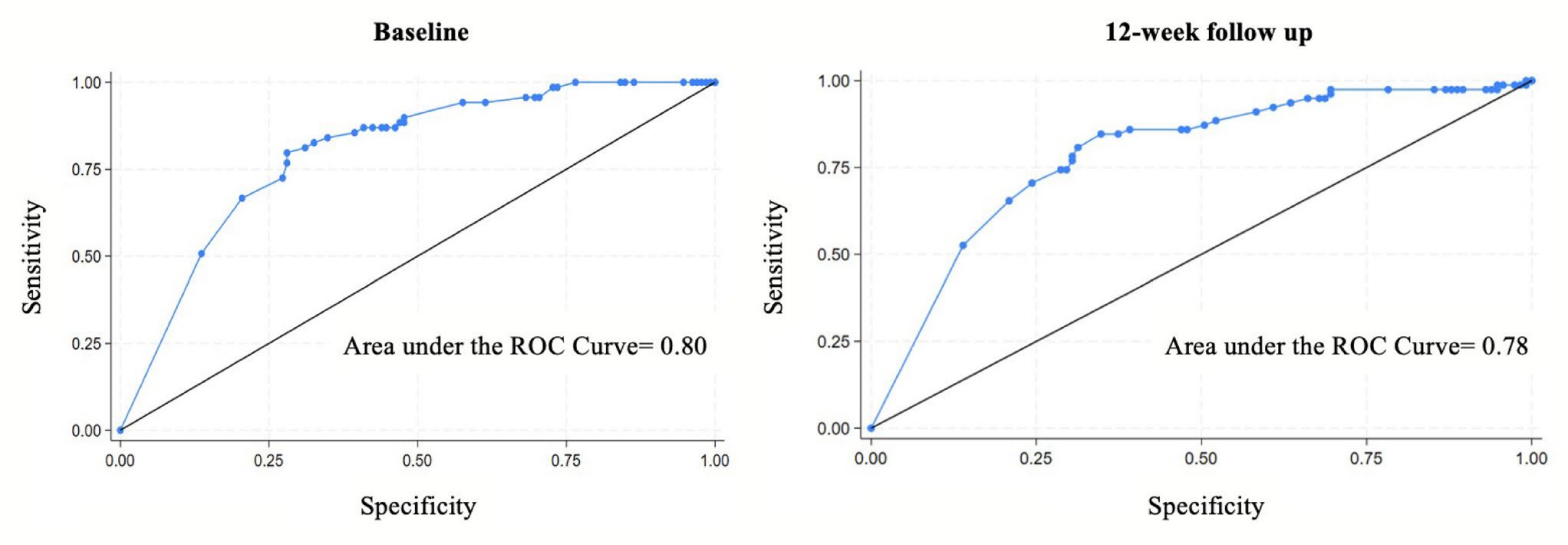
ROC Curves demonstrating discriminatory power of the EQ-5D-3L utility values to detect poor HRQOL in people with aphasia at two time points

ROC curves were used to explore how well the EQ-5D-3L utility values and VAS could classify people with aphasia according to their self-rated HRQOL on the SAQOL-39g (Fig. 3). The sizes of the AUC were calculated as exploratory analyses. Results suggested the EQ-5D-3L demonstrated reasonable classification performance when distinguishing participants with lower HRQOL (AUC = 0.80 at baseline, 0.78 at 12 week follow up), as defined by total mean scores of four or below on the SAQOL-39g. The VAS showed moderate classification performance at baseline (0.78), and more limited performance at 12-week follow up (0.69; Supplementary Fig. S1).

### Factor analysis

Factor analysis identified two distinct factors with eigenvalues above 1 at both time points. At baseline, factor 1 explained 45% of variance (eigenvalue 3.16), and factor 2 explained 19% of variance (eigenvalue 1.33). Factor 1 was characterised by loadings from the SAQOL-39g physical domain in addition to the EQ-5D-3L mobility, self-care, and usual activities domains. Factor 2 included the SAQOL-39g psychosocial and communication domains, and the EQ-5D-3L pain/discomfort and anxiety/depression domains (Table 4). At 12 weeks, factor 1 explained 27% of variance (eigenvalue 1.89), and factor 2 explained 20% (eigenvalue 1.39). The factor structure was relatively stable at the two time points. Collectively, the instruments demonstrated some overlapping constructs (physical functioning and psychosocial well-being), confirming the construct validity of both measures. However, each instrument captured a unique domain of HRQOL: the EQ-5D-3L uniquely measured pain and discomfort (uniqueness 0.66), while the SAQOL-39g uniquely measured communication (uniqueness 0.73). These findings indicate that communication-related HRQOL, as captured by the SAQOL-39g, would not be adequately assessed using the EQ-5D-3L alone in people with aphasia.

**Table 4:** Factor analysis of SAQOL-39g and EQ-5D-3 level domains at baseline and 12-week follow up

Domains	Baseline			12-week follow up		
	Factor 1	Factor 2	Uniqueness	Factor 1	Factor 2	Uniqueness
**EQ-5D-3L** ^*^						
Mobility	**0.58**	0.28	0.57	**0.59**	0.12	0.62
Self-care	**0.72**	0.01	0.47	**0.63**	0.06	0.58
Usual activities	**0.54**	0.23	0.64	**0.54**	0.15	0.67
Pain/discomfort	0.31	**0.48**	0.66	**0.46**	0.21	0.73
Anxiety/depression	0.22	**0.49**	0.70	0.14	**0.52**	0.70
**SAQOL-39g** ^*+*^						
Physical	**0.73**	0.33	0.34	**0.72**	0.37	0.33
Psychosocial	0.20	**0.68**	0.48	0.25	**0.71**	0.41
Communication	0.18	**0.47**	0.73	0.12	**0.60**	0.61

*Factor loadings*: < 0.3 poor, 0.3–0.49 fair, 0.5–0.59 good, 0.6–0.69 very good, ≥ 0.7 excellent

^*^ *EQ-5D-3L: Euroqol EQ-5D 3 Level.*

^*+*^*SAQOL-39g: Stroke and Aphasia Quality of Life scale*,* 39 item. SAQOL-39g domain scores were reversed for consistency of severity direction between the two measures*

## Discussion

To our knowledge this is the first study to compare self-rated HRQOL scores between a generic and aphasia-specific HRQOL instrument. Our findings provide evidence that EQ-5D-3L utility values established with an Australian algorithm effectively discriminate between people with aphasia who have poor quality of life, and those who do not. However, the EQ-5D-3L demonstrated limitations when compared to the SAQOL-39g, including ceiling effects, weak domain correlations and low construct validity. Together, the findings highlight important constraints about the EQ-5D-3L’s utility values adequately reflecting the HRQOL impacts experienced by people with chronic mild or moderate aphasia. Since a large proportion of patients with stroke experience aphasia, there is a need to evaluate alternative patient-reported outcome measures that can produce utility values for economic evaluations. Potential alternatives include the Assessment of Quality of Life (AQoL), the Health Utilities Index Mark 3 (HUI3) and the Quality of Life Questionnaire 15 Dimensions (15D) [25–27]. Each of these instruments include an item measuring communication-related quality of life but have yet to be evaluated in this population. Such evaluation could help ensure prioritisation of high-value treatments in aphasia care.

The EQ-5D has been used in five cost-utility analyses of aphasia treatments to date, using the 3L version [21, 28], 5 L version [29, 30], or unspecified version [31]. Treatment types were varied, including computerised word finding [29, 31], transitional support [28], peer befriending [30], usual care, constraint-induced therapy, and multi-modality therapy (data source for current study) [21]. In four of these analyses, the EQ-5D and VAS were modified to increase accessibility through aphasia-friendly formatting (increased spacing, bold key words, and pictures) [29–31]. However, the domains of the EQ-5D remained unchanged [5, 29–31]. Whether the EQ-5D-3L can capture the full impact of a health condition on HRQOL has been critiqued internationally, and across health conditions [32]. Limitations in communication activity and participation can have profound impacts on HRQOL for people with aphasia [3, 4, 33]. Therefore, the absence of a communication domain on the EQ-5D-3L raises concerns about its suitability for informing economic decision making in this patient population [3, 6]. Addition of a communication dimension (or ‘bolt-on’) to the standard EQ-5D may be a practical solution to enhance instrument’s relevance for people with aphasia [34].

The VAS is intended to supplement the EQ-5D-3L utility values; however, has been used as a stand-alone measure in 14 economic evaluations [35]. Despite demonstrating a moderate correlation with the SAQOL-39g total score at both time points, our ROC analyses indicate the VAS exhibits poor to acceptable discriminative ability in measuring poor quality of life in people with aphasia. Consequently, use of the VAS as a substitute for utility values in economic evaluations which include people with aphasia is not recommended.

The distinct instrument designs of the EQ-5D-3L and SAQOL-39g do not capture patient health states in the same way. The SAQOL-39g has 39 items, compared to 5 on the EQ-5D-3L. This means that multiple SAQOL-39g items overlap conceptually with items across the EQ-5D-3L (e.g. walking, balance, standing, climbing stairs overlap with mobility) while other SAQOL-39g items (e.g. confidence, fatigue) are not directly assessed on the EQ-5D-3L [9, 15]. The SAQOL-39g communication domain comprises seven specific questions which seek to understand the recognised impact of communication impairment on HRQOL post-stroke, while the EQ-5D-3L does not directly assess communication-related HRQOL [36, 37].

Our analysis revealed distinct measurement properties between the instruments, despite theoretical expectations of overlap. It was theorised that the EQ-5D-3L usual activities domain, which asks people to describe their usual activities such as work or study, may elicit a comparable rating to communication-specific items on the SAQOL-39g, since these activities involve various forms of communication [9]. Yet, in the present study, the domains of the two instruments demonstrated a consistently poor relationship at both time points (Tables 3 and 4). Factor analyses confirmed this distinction by revealing two distinct domains, with communication a domain unique to the SAQOL-39g. (Table 4), This demonstrates that the instruments measure unique elements of HRQOL with evident discriminant validity between dissimilar dimensions. These findings should be interpreted cautiously, given the potential impact of differing response scale ranges on correlation coefficients and the variability in correlation strength classification systems across the literature [23].

The score differences between the EQ-5D-3L and the SAQOL-39g in the current study may also reflect differences in instrument design. In their think-aloud study, Engel et al. identified challenges completing the EQ-5D among aged care residents, including unclear instructions leading to multiple responses per item (*n* = 5) and inadequate response options for items with two constructs, such as anxiety/depression [38]. Similar completion challenges with the EQ-5D have also been raised by people with asthma, learning disabilities, and healthy individuals [39–42]. Given that people with aphasia can have difficulty understanding abstract concepts, the EQ-5D’s format may present additional challenges [43]. For example, the mobility item requires respondents to understand that walking-related statements represent broader mobility concepts. In contrast, the SAQOL-39g uses concrete, specific questions following a predictable structure (“how much trouble did you have…” followed by specific activities like walking, keeping your balance, or climbing stairs) [15]. As a condition-specific instrument, this clear and concrete approach to item design maybe more accessible than the shorter, yet more abstract EQ-5D instrument. This hypothesis supports the importance of population-specific validation before using HRQOL instruments in economic evaluations.

We observed ceiling effects on the EQ-5D-3L, a well-documented limitation of this instrument [44–47]. In the current study, between 45 and 79% of the participants rated themselves as having ‘no problems’ across the HRQOL domains at baseline. By comparison, 0–6% of participants rated themselves as having ‘no trouble at all’ across the SAQOL-39g domains. These results are consistent with the minimal ceiling effects reported in SAQOL-39g’s psychometric testing [15]. Ceiling effects may lead to unintended consequences for economic evaluations; a patient’s health state may be over-estimated by selecting ‘no problems’ if a response option that better represented their HRQOL was not presented. In the resulting economic evaluation, the cost-effectiveness estimate may be biased, as the effectiveness of an intervention may be underestimated. Additionally, ceiling effects may limit the ability of the EQ-5D-3L to demonstrate the benefit of a treatment in people with less severe aphasia [46]. The EQ-5D instrument has been further developed to include five response levels. A substantial reduction in ceiling effect has been observed in a general English-speaking population compared to the EQ-5D-3L, however, in their systematic review, Feng and colleagues reported that the 5-Level instrument was not correlated with communication measures [48]. Therefore, while the additional response levels may overcome the ceiling effects in the current study, use of the EQ-5D-5L in its current form does not address the concerns about conceptual appropriateness for people with post-stroke aphasia.

Altogether, the EQ-5D-3L and SAQOL-39g provide two conceptually different methods for patients to describe their health state. Despite uncertainty regarding which of the specific factors (response options or content) contributed to the response patterns, the result was two distinct distributions of HRQOL scores. These findings suggest use of the EQ-5D-3L in economic evaluations including people with aphasia warrants careful consideration. With up to 40% of people with stroke experiencing aphasia, the EQ-5D-3L instrument may underestimate the overall burden of stroke. Therefore, inaccurate measurement of HRQOL in people with aphasia has implications for economic evaluations comparing stroke outcomes with other health conditions.

### Strengths and limitations

This study provides novel evidence comparing the structure and performance between a generic and an aphasia-specific HRQOL instrument. The standardised collection of data within a large-scale randomised control trial is a further strength. Although these findings are important for guiding the future direction for economic evaluations of aphasia treatment, it was a preliminary exploration of an established dataset. Therefore, patient perceptions of EQ-5D-3L acceptability and comprehensibility were not investigated. The study population comprised primarily participants with mild to moderate chronic aphasia after stroke, which may present limitations in generalising our findings to people with more severe aphasia Additional analyses comparing responses between participants with mild and severe aphasia were not possible with the small sample of people with severe aphasia (*n* = 6).The algorithm used to convert EQ-5D-3L domain scores to utility values was validated in an Australian-representative sample. Algorithms converting EQ-5D scores to utility values have significant influence on whether the resulting utility values are meaningful. Research has demonstrated substantial cross-country variation in EQ-5D-3L utility values, depending on the valuation method used. Therefore, while our use of an Australian algorithm was appropriate for this study population, the resulting utility values may not be generalisable to other countries with different health state preferences [49]. Results from patients with aphasia after an acute stroke, or converted using a different valuation algorithm, may have also produced different results.

## Conclusion

In its current form, the EQ-5D-3L demonstrates poor convergent and construct validity when compared to the aphasia-specific SAQOL-39g. Additionally, ceiling effects were present when measuring HRQOL in a population with primarily mild and moderate, chronic aphasia. These findings raise questions about the appropriateness of using the EQ-5D-3L to conduct economic evaluations that include people with aphasia. As neither the EQ-5D-3L nor the SAQOL-39g can reliably generate utility values reflecting the health states of people with aphasia, evaluation of alternative HRQOL instruments in this population is required to ensure the highest value aphasia treatments are prioritised in clinical care.

## Electronic supplementary material

Below is the link to the electronic supplementary material.

Supplementary Material 1

Supplementary Material 2

## Abbreviations

HRQOL: Health related quality of life
QALY: Quality adjusted life year
EQ-5D-3L: Euroqol 5 dimensions health questionnaire, 3 level
EQ-5D-5L: Euroqol 5 dimensions health questionnaire, 5 level
SAQOL-39g: Stroke and aphasia quality of life scale, 39 item
VAS: Visual analogue scale
