# Supplementary material for: Aphasia-specific or generic outcomes? a comparison of two health-related quality of life instruments for economic evaluations of aphasia treatments
Source: Qual Life Res. 2025 Jul 26;34(10):2879–90. doi: 10.1007/s11136-025-04040-8 (PMC12535536; doi:10.1007/s11136-025-04040-8)
Supplement: Supplementary file 2 — Supplementary Material 2 [file 11136_2025_4040_MOESM2_ESM.docx]

**Supplementary Figure 1: ROC Curves demonstrating discriminatory power of the EQ-5D-3L visual analogue scale to detect poor HRQOL in people with aphasia at two time points.**

**
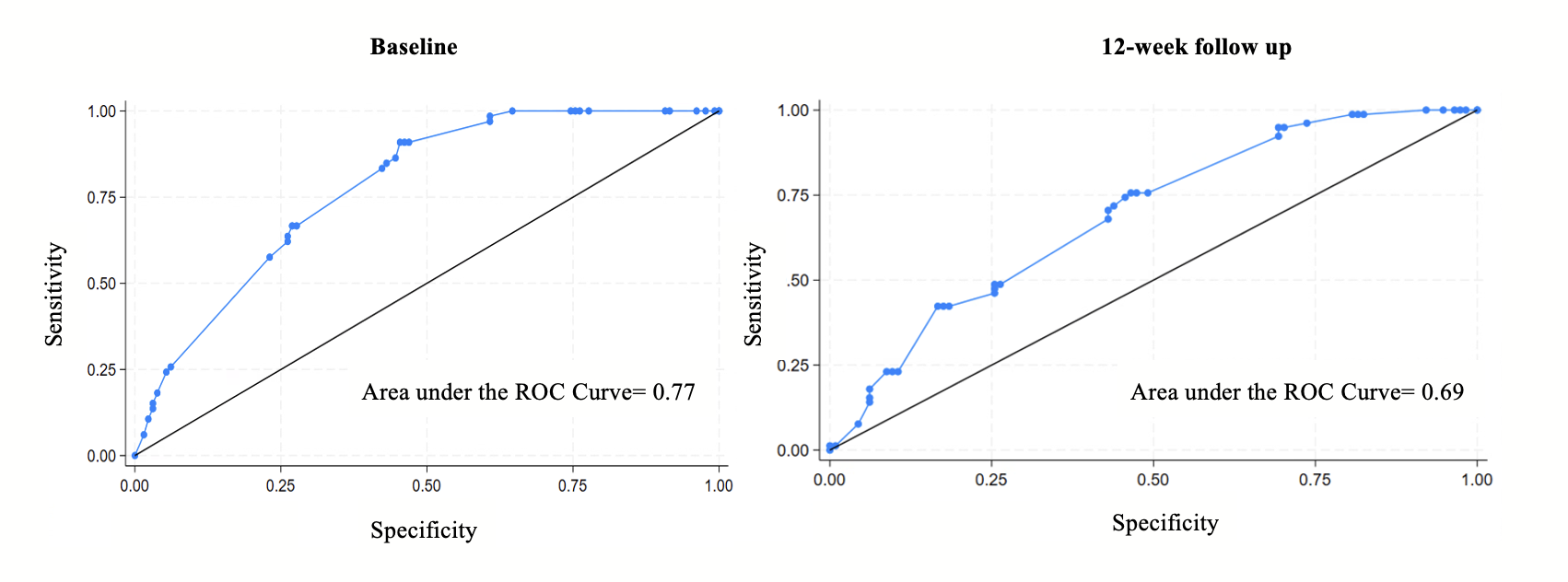
**
